# Abstracts and Gleanings

**Published:** 1884-12-20

**Authors:** 


### ABSTRACTS AND GLEANINGS.

**Case of Dropsy Cured.**—A case is reported in the New England Medical Monthly of "a woman who did not seem to regain her strength after her last confinement. Dropsy set in ; both legs were very œdematous ; breathing very short and difficult. The lips cyanosed, and pulse extremely weak ;" the urine I did not examine, being sufficiently satisfied with ocular general inspection, ausculation, etc.

Besides œdema of the legs, there were ascites, hydro pericardium and hydro-thorax. Something had to be done quickly ; aspiration and punctures not being indicated as measures affording permanent relief, I exhibited one pill of elaterium, containing one-sixth grain. The patient was in such a weak condition, and apparently so near death, that some advisers would have doubted the propriety of exhibiting such a powerful drug as elaterium. In the administration of medicines, as well as in operative procedures, advisers very properly are swayed often in their judgment by concomitant circumstances ; still there confront us ever and anon instances where we have to act rapidly and in a contradictory manner ; such acts as these latter are chronicled of locomotive engineers. The woman in question was of large frame and muscular ; if she had been a "delicate woman" I think I should have hesitated in administering the elaterium. The drug acted violently, by emesis and catharsis ; the next day she was well, the four dropsies having disappeared. She kept well for four years—that is, had no return of dropsy. She then had the same collection of dropsies as at the first ; the same treatment dissipated the water. Two years afterwards the dropsies reformed ; the same treatment was, as usual, effective. In another year dropsy returned ; I was at the time disabled from attending to my patients ; instructions were not properly carried out, and she died.

**Experiences with Hydrochlorate of Cocoaine.**—Immediately after the publication of the experience of Drs. Agnew and Noyes with hydrochlorate of cocoaine I obtained some of the drug and tried it in 3 per cent. solution on the following cases :

1st. ACUTE CATARRH.—A few drops were introduced by the finger and camel's hair pencil into the nasal cavity at intervals of five minutes, and at the end of fifteen or twenty minutes considerable relief was experienced, accompanied with a certain amount of anæsthesia.

CASE 2. Some of the same solution was gently rubbed into herpes of the upper lip, producing numbness resembling tinct. of aconite but unaccompanied with any burning sensation. At the end of twelve minutes superficial pinching produced no pain, but the deeper parts of the lip retained normal sensibility.

CASE 3. Inflamed, protruding hemorrhoids were bathed, at intervals of five minutes, with a little of the solution, and at the end of twenty minutes could be handled without producing pain.

CASE 4. Three or four drops were rubbed by the finger at five minute intervals upon the mucous membrane of the mouth, around a tooth that was about to be extracted, producing anæsthesia of the gum, but not causing any effect upon the deep seated pain as the tooth was drawn from its socket.

CASE 5. A hard, painful corn was bathed with some of the same solution, but, owing to the thickness of the tissue, no appreciable effect was produced.

CASE 6. A painful scirrhus of the breast (with the skin unbroken) was bathed with drops of the solution for fiteen minutes, but no appreciable effect seemed to ensue.

From the above it would appear that hydrochlorate of cocaine will be of very great service to mitigate, or entirely stop, pain in mucous membranes or where small operations have to be performed ; but, from the superficiality of its action, it is not suitable where skin or deep structures have to be extensively destroyed. The class of cases in which it will prove most useful are irritations of the eye, throat, mouth, vagina, rectum, and uterus, and for examinations during labor, as well as in its last stages, and for vaginismus and prurigo it will be invaluable.—*Dr. Uhler, at Baltimore Acad. Medicine.*

**The Effects of Bromoform, Bromethyl, and Bromethyline.**—Bonome and Massa, from a series of physiological experiments recently conducted in the laboratory of Prof. P. Albertoni, in the University at Genoa, obtained the following results :

1. Bromoform is a general anæsthetic. Dogs and Guinea-pigs almost always show the same symptoms of anæsthesia and muscular relaxation, following inhalation, that the human subject does. In five experiments upon men, three were well narcotized, lasting even for a whole hour ; in two (probably on account of the use of a defective preparation containing free bromine) there was no narcosis, but, on the contrary, irritation of the conjunctivæ, a flow of tears, burning in the eyes, etc. The narcotic action they believe to be a little slower in appearing than when chloroform or ether is used, but the success is apparently the same as with both of these valuable anæsthetics.

2. The narcosis obtained from the inhalation of bromoform is free from the stage of exaltation which we are called upon to witness during chloroform-administration. On this account preference should be given this agent in case the patient is subject to epilepsy or alcoholism. Billroth and Nussbaum have each directed attention to the danger of exciting attacks in epileptics by the use of chloroform. This does not occur during the use of bromoform, which allays the irritability of the cerebral cortex.

3. Bromoform does not disturb the respiratory function, but after prolonged narcosis there is a slight reduction of the blood-pressure. The respiratory fluctuations of the blood-pressure in the course of the narcosis are very regular ; the pulse remains strong. In none of the dogs which inhaled it did the bromoform cause sudden arrest of the heart's action, such as is seen during the use of chloroform.

4. During the bromoform narcosis, while it was noticed in dogs that there was decided mydriasis, in man there occurred only trifling alterations of the pupil ; there was neither nausea or vomiting. The quantity of bromoform required to produce complete narcosis is less than that of chloroform, as it is commonly used.

5. In the first few hours after narcosis, it was noticed that there was a sinking of the temperature exactly as after chloroform, but the patient appeared to recover sooner from its effects.

6. Given by the mouth, bromoform also acts as a hypnotic and anæsthetic.

7. Moreover, bromoform prevents putrefaction in organic substances (urine and meat) ; bacteria are not developed in the presence of bromoform.

8. Injected under the skin, bromoform is fatal when given in a dose of 0.15 gm. for every 100 gm. of the bodily weight.

Ethyl bromide produces narcosis more quickly than chloroform or bromoform, but is more easily eliminated from the system, and, on this account, its effects are more temporary. It is to be recommended for short operations. It is less active than bromoform, and becomes poisonous at a point of each 0.17 gm. for 100 gm. of the bodily weight. Whilst the narcosis reduces the blood-pressure (20-30 mm.) at first, it rapidly increases again after the termination of the narcosis, when the respiration also is accelerated. Bromide of ethyl also reduces the irritability of the cerebral cortex, and likewise hinders the development of bacteria in organic infusions.

Ethylene bromide does not produce complete narcosis upon inhalation, but, when pushed, causes fatal results by abolishing the cardiac activity.—*Centralblatt fur Chirurgie, No. 36, Sept. 6.—Medical Times.*

**Some Statistics of Abdominal Surgery.** — For the greater part of this compilation, we are indebted to a report presented at the last meeting of the Medical Society of North Carolina, by Dr. Staton, chairman of the section on Surgery :

*Gastrotomy for Myofibromata of the Uterus.*—The extracts just below are taken from Professor Bigelow's paper, published in the American Journal of Obstetrics, November and December, 1883.

The tumor alone removed 106 times; 30 recovered; 50 died. The tumor and uterus removed 229 times ; 124 recovered ; 94 died. Extra-peritoneal cases, 247 times ; 143 recovered ; 97 died. Intra-peritoneal cases, 84 cases ; 50 recovered ; 33 died.

Bantock's individual record shows 22 cases, with 2 deaths. Hegar records 12 cases, with 1 death. By the intra-peritoneal operation, Schroeder lost only 1 in 14. However, general statistics favor the extra-peritoneal method of operating.

*Vaginal Hysterectomy.*—Statistics : Fenger, 45 cases ; 31 recovered ; 3 unfavorable ; 11 deaths. Schwaltz, 55 cases ; 35 recovered ; 20 deaths.

*Abdominal Hysterectomy.*—Statistics: Ahlfed, 66 cases; 49 deaths Kleinwachter, 94 cases ; 20 recoveries. This operation is taking precedence in popular favor.

*Cæsarian Section.*—The following figures are taken from the valuable statistics of this operation, prepared by Dr. Harris, of Philadelphia:

The Cæsarian Section has been performed in North America 133 times—the first in 1827; 60 women saved: in Great Britain, 134 times; 24 women and 78 children saved. Bruden, in 1873, wrote that during a space of eighty years, 50 C. sections were made in Paris—all fatal. The Porro modification of the C. section has saved 46 plus per cent. The Porro-Muller method has saved 52 plus per cent.

[The statistics of Dr. Harris, published in the American Journal of Medical Sciences, April, 1878, contain the record of 9 cases of C. section performed in Louisiana; 8 mothers were saved; 1 died; 5 children survived; 3 died; 1 died of the effects of a long labor, 2 of craniotomy. In 1838, on a river plantation near New Orleans, a very remarkable Cæsarian section was performed by a drunken negress, in the case of a young black girl in her first labor. There was no obstruction to the labor. However, the operation was performed with a sharp case-knife, and a living child removed. The mother made a good recovery, save slight incontinence of urine. The case was reported by Dr. Bennett Dowler in the July (1854) number of this journal.]

*Oophorectomy.*—Abdominal oophorectomy is now taking precedence over the original operation, and, in most of the cases of his last series published, was practiced by Battey himself. In either of these operations the mortality is about 18 per cent.

*Ovaria-Salpingectomy.*—The mortality of this operation has been 77 per cent.

*Gastrectomy.*—This operation, which usually consists of a resection of the pylorus, was first performed by Pean, in 1879. It has since been performed 16 times by Billroth; 12 died; 4 recovered.

*Operations on Small Intestines.*—Ashurst has tabulated 13 cases of intususception, treated by abdominal section, of which 5 recovered. Four of the cases occurred in children; all died. Of 57 cases of acute intestinal obstructions from other causes, 18 recovered after abdominal section.

*Nephrectomy.*—Gross has recorded 104 cases of nephrectomy; 52 recoveries, 46 deaths; 6 still under treatment. Of 24 cases of removal of the healthy kidney, 18 recovered; 6 died.

[On the 3d of June, 1879, Dr. Smyth, of New Orleans, made a successful nephrectomy in a case of floating kidney. The report is published in the August (1879) number of this journal.]

*Splenotomy.*—The operation of cutting off a part of the organ has been performed about 30 times, with a mortality of 50 per cent.

*Splenectomy.*—Extirpation of the spleen has been performed in 37 cases; 7 recovered. Of 19 cases of leucocythemic spleens, 18 died. Of 11 cases of simple hypertrophy, 6 recovered.—*New Orleans Med. and Surg. Journal.*

**Foreign Bodies in the Air Passages.**—Dr. J. R. Wiest, after a study of one thousand cases of foreign body in the air passages, reaches these conclusions, and submits them to the profession for consideration:

1. When a foreign body is lodged either in the larynx, trachea, or bronchi, the use of emetics or similar means should not be employed, as they increase the suffering of patient and do not increase his chances of recovery.

2. Inversion of the body and succession are dangerous and should not be practised unless the wind-pipe has been previously opened.

3. The presence simply of a foreign body in the larynx, trachea, or bronchi does not make bronchotomy necessary.

4. While a foreign body causes no dangerous symptoms, bronchotomy should not be performed.

5. When a foreign body remains fixed in the trachea or bronchi, as a general rule, bronchotomy should not be practised.

6. When symptoms of suffocation are present, or occur at frequent intervals, bronchotomy should be resorted to without delay.

7. When the foreign body is lodged in the larynx, there being no paroxysms of strangulation, but an increased difficulty of respiration from œdema or inflammation, bronchotomy is demanded.

8. When the foreign body is movable in the trachea and excites frequent attacks of strangulation, bronchotomy should be performed.—*Weekly Med. Review.*

**Substitute for Veratrum.**—Dr. Brantley, in the Atlanta Medical Journal, writes : " Now, after long years of observation, I come to venture a substitute for veratrum, which has called forth this communication—that *brandy* is the *remedy.* I have to say that when properly administered it is the safest, surest, and most effectual remedy ; in doses from 6 to 30 drops, given regularly every two hours, it will almost invariably reduce the pulse in a few hours from 120 to 80. Its effects are so marvelous that I was long in accepting the results from the use of the spirits. It appears to supply the system with the necessary carbon, and in a great measure relieves the tissues and supplies material for the disease to prey upon. Although prejudiced in the extreme to alcohol, I am forced to make terms with it in this ailment, and I think that it is rarely necessary to give over 10-drop doses ; its use indiscriminately doubtless has often proved worse than useless in the treatment of typhoid fever. The tympanites so often complained of, and for which turpentine and many other nauseating remedies, together with blisters, have so often been used, is ordinarily nothing but wind or gas in the large bowels, and can be expelled and prevented by carminitives."

**Hemorrhoids, Dilatation of Anus for.**—Dr. Verneuil (Medical Review) was called by one of his confreres to see a gentleman who had been suffering excruciating agony for two days from strangulated piles, and on whom ice fomentations, narcotics and other means were tried without effect. Local examination showed no more than is ordinary in such cases—a ring of tumefied external hemorrhoids, with a dark spot in the center, announcing the commencing of sphacelus. The least touch was painful, and the patient demanded relief at any price. Partisan of the treatment of hemorrhoids in general by dilatation, he thought that he would be doing right in employing it in the present case, knowing that, by suppressing the general action of the sphincter, the pain wouid cease. Accordingly, the patient was put under the influence of chloform, and the hemorrhoidal tumors reduced, and M. Monod largely dilated the anus with his fingers. A few minutes afterwards the patient awoke free from all pain, and in a few days he had the satisfaction of not only feeling that the strangulation had entirely disappeared, but that he was forevea quit of the piles. This case of Monod's proves that the hand dilates just as well as the speculum, and consequently the operation is reduced to its simplest expression.

### MY CHILD'S ORIGIN.

One night, as old Saint Peter slept,
He left the door of Heaven ajar,
When through a little Angel crept,
And came down with a falling star.

One summer, as the blessed beams
Of morn approached, my blushing bride
Awakened from some pleasing dreams,
And found that Angel by her side!

God grant but this—I ask no more—
That when he leaves this world of pain
He'll wing his way to that bright shore,
And find his way to Heaven again!

### SAINT PETER'S REPLY.

Full eighteen hundred years, or more,
I've kept my gate securely tyled;
There was no "little Angel" strayed,
Nor one been missing all the while.

I did not sleep, as you supposed,
Nor left the gate of Heaven ajar,
Nor has a "little Angel" left
And gone "down with a falling star!"

Go ask that "blushing bride," and see
If she don't frankly own, and say,
That when she found that "Angel" babe,
She found it in the GOOD OLD WAY!

"God grant but this—I ask no more"—
That should your numbers still enlarge,
You'll not do as you did before,
And lay it to Old Peter's charge!

**The Dry Diet Treatment of Ulcer of the Stomach.**—M. Debove, in Le Progres Medical, points out certain risks in the ordinary milk treatment of ulcer of the stomach. The ingestion of so much fluid, viz, five to seven pints of milk in the twenty-four hours, into a stomach with atonic mucous membrane, is apt to produce almost fatal dilatation, and the stretching of the ulcer to provoke hæmorrhage and perforation. Indeed, all excessive dilatation of the stomach is dangerous, while contraction is favorable. M.

Bouchard, for a long time, in this disease, has given a dry diet, consisting of finely divided nutritious food—such as meat powders—with not more than three-quarters of a part of pure water, during the twenty-four hours ; and, under this regimen, he has seen the morbid dilatation of the stomach diminish or disappear. M. Debove's plan is as follows : The stomach is first carefully washed out, but the operation is to be instantly stopped if the returning liquid is tinged with blood. Then the patient is to take three meals daily, each consisting of meat powder, 25 grammes ; bicarb. of soda, 10 grammes, and, as the mixture is not very palitable, it is well to introduce it into the stomach by a siphon. To this dry food, which represents 300 grammes (about ten ounces) and 30 grammes (about one ounce) of sod. bicarb. per diem, 1 litre (nearly one quart) of milk is added, which is to be drunk in small quantities at a time. M. Debove's object is to annul the action of the gastric juice, and thus stop digestion and favor the healing of the ulcer.

The albuminoids are not converted into peptones in the stomach, and their alkiline reaction is very favorable to their digestion in the intestine. Perhaps, too, the soda is directly beneficial to the ulcer. Two severe cases of gastric ulcer, accompanied with very painful crises, by hæmorrhage, and by great emaciation, which had resisted all treatment, were quickly cured by the above treatment of Dr. Debove.—*Medical Chronicle.*

Professor Da Costa finds sulphate of copper, gr. 1-12–1-8, four times a day, combined with opium, to be very effective in chronic dysentery. Other medicines he finds useful are bismuth (gr. x, the adult dose being three or four times a day), especially in children, nitro-hydrochloric acid, zinc sulphate, argentic nitrate, iron sulphate of Monsell's solution (gtt. iij-v.), or solution of the nitrate (gtt. xx-xxx). All, except iron, should be combined with opium. When other things fail, small blisters over the spot of greatest soreness sometimes do good. The diet should contain no starches, fruit or other vegetables.—*Peoria Med. Monthly.*

**The Medicines Physicians Use.**—Squibb's Ephemeris gives an analysis, containing some points of interest, of some observations made by Dr. William P. Bolles on the prescriptions which he found on the files of three Boston pharmacists. The number counted was 3,726, which were pretty generally from physicians of that city. The number of articles entering these prescriptions was 504, the whole number contained in the U. S. P. for 1880 being 994. Of the 504, 236 occurred 5 or more times ; 157, 10 times ; 80, 25 times; 27, 50 times ; 9, 100 times ; 1, 200 times. Sulphate of quinine leads the list, and is found in 292 of the 3,726 prescriptions ; sulphate of morphine in 172; bromide of potassium in 171; iodide of potassium in 155 ; tincture of chloride of iron, 134; subnitrate of bismuth, 133; glycerin and syrup taken, 120 ; syrup, 108 ; carbolic acid, 92 ; extract nux vomica, 87 ; paregoric, 80 ; bicarbonate of soda, 77 ; calomel, 72 ; chlorate of potassium, 71 ; compound tincture of gentian, 67 ; lime water, 65 ; and so on down. It will be thus seen that of 994 articles of the Pharmacopœia only 18 occur more than 65 times in 3,726 prescriptions, and of these 17 three are vehicles or adjuncts which are in such common use as to bring their numbers into prominence. Dr. Squibb regards it as surplusage of a very useless kind to have a drug in substance, in abstract, decoction, infusion, extract, fluid extract, and tincture. He says the individual habits of physicians are the cause of much of this surplusage. One of the remedies for this evil he points out as follows: "The individual preferences of physicians are largely prejudices adopted from teachers in the schools, and, therefore, if the schools would but reason upon the subject, and direct only the best preparation of each drug, a needed reform in the Pharmacopœia would soon follow, and the pharmacist's supplies would be much fresher and more trustworthy.—*Medical Age.*

**Crede's Method of Expression of the Placenta.**—In a paper, which is a reply to certain objections to his method, by Ahlfeld, Crete (Abender gegen Ahlfeld's Berichte und Arbeiten, etc.) gives a very clear description of the mode in which his method is to be earried out (Edin. Clin. J.): "Immediately after the birth of the child, the uterus must be controlled by the hand laid on it, and the third stage shortened in this way, that the natural contractions of the uterus are increased by gentle, soft friction by the hand, and the contractions, which would otherwise be delayed, are excited. As soon as the hand, resting on the uterus, feels a more powerful contraction, with which some blood escapes, and the uterus becomes distinctly smaller in the hand, the uterus must be grasped on all sides with the fingers of one or both hands, and with the fundus directed forwards and upwards, *gently* pressed downwards toward the hollow of the sactum, rather towards the coccyx, i. e. with the patient lying horizontal on her back—vertically downwards into the small pelvis. This pressure is only to be made during the acme of a pain." The placenta will, as a rule, be expelled with third or fourth contraction, i. e. five minutes or more after the expulsion of the child. If not expelled then, we muet delay expression till the contractions are renewed.—*Jour. Am. Med. Association.*

**Numbness of Upper Extremities.**—In a paper, read before the College of Physicians, Philadelphia, Dr. Wharton Sinkler calls attention to a form of numbness, especially of the upper extremities, occurring usually in women at about the change of life, though he has found the same condition occasionally in men.

The numbness generally begins in one or both hands and gradually extends up the arms. It is almost always worse in the morning before the patient rises. It is described as a "tingling," or a sensation "pins and needles," or as if the limb "were asleep."

There is generally a transient weakness, but no paralysis; often pain, and sometimes a sensation as if the numb part were swollen. Dr. Sinkler thinks that there is probably a hyperemia of the nerve trunk or a part of the cord, and he notes the fact that the recumbent position at night seems to increase the numbness as a confirmation of this view, as the supine position favors an increase of blood in the cord and nerves of the extremities.

As to treatment, he has derived the most satisfaction from the administration of ergot. Massage and galvanism have been valuable adjuvants, and bromide of potassium was helpful in some cases. He found the use of strychnia, for a time after the disappearance of the numbness, to be of great advantage.– *St. Louis Courier of Medicine.*

**Salicylic Acid and Spirits Nitre as a Febrifuge.**—The following formula used by Dr. J. P. Thomas during the exascerbation of fever, would be useful also in rheumatism :

| | | |
|---|---|---|
| ℞. | Salicylic acid | 2 drachms |
| | Sweet spirits nitre | 6 ounces. |

Mix. Dose, a tablespoonful, diluted, every two hours. For children under twelve years, two teaspoonsful ; infants, from a half to a teaspoonful. Have found this treatment very satisfactory.

Dr. F. M. Jones, in the Medical World, says : I send you my favorite prescription for piles :

| | | |
|---|---|---|
| ℞. | Bismuth subnitrate | gr. 30 |
| | Powdered hydrastis | dr. 1 |
| | Vaseline | oz. 1 |

First moisten bismuth and hydrastis with glycerine, and then add vaseline.

Sig.. Apply two or three times a day with finger.

**Emenagogue.**—Dr. Calver (in the Medical World) recommends the following emanagogue : In cases of retention, I frequently give the following :

| | | |
|---|---|---|
| ℞. | Sat. Tict. Cimifuga Rac. | ʒ j |
| | Aqua | ℥ iv |

M. S.: A teaspoonful every two or three hours ; commencing a week before the menstrual epoch. If the case is one likely to be tardy, and require increased doses, I usually, on the day before, or on the day the discharge is due, increase to a drachm of the tincture three times daily.

**A Decoction of Lemon as a Cure for Ague.**—As a popular remedy the use of lemon has had considerable influence among the laity in certain parts of the world.

Lately it has been brought into prominence by Dr. Conrad T. Crudelia, Professor of Hygiene at the University of Rome, Italy. In his address before the Copenhagen International Medical Congress, he tells us that the decoction is prepared by cutting up one lemon, peel and all, into thin slices, which are placed in three tumblers of water, and the whole boiled down to one glassful. It is then strained through linen, squeezing the remains of the boiled lemon, and set aside for some hours to cool. The whole amount of the liquid is then taken, fasting. Lemon juice, or a decoction of lemon seeds, are used in Greece and North Africa as a remedy for malarial fevers of moderate intensity. In Guadaloupe a decoction of the bark from the root of the lemon tree is used for the same purpose. These popular practices would seem to indicate that there is some antimalarial virtue in the lemon tree. While it is useful in acute malarial disease, it is especially valuable in chronic cases.

The doctor, being convinced that the remedy had a positive value, persuaded some proprietors in the Roman Campagna to try it with their employees. The good effects here were conclusive. From these he gradually persuaded practitioners to test the treatment. The results of these quite extensive observations confirmed his first experience.

In the interests of humanity we shall hope that farther observations shall confirm the anticipations of the distinguished Roman professor. If decoction of lemons will cure malarial poisoning it will be relief to the cinchona trees.—*Detroit Lancet.*

**Pieces of Glass Pass Through the Digestive Tract.**—Dr. L. R. Markley writes to the editor of the Medical World :

Sunday evening, November 9, 1884, I was called to see the child of Mr. B., aged one year and ten days.

When the child was in a room by itself it pulled the tube from its nursing bottle and chewed off quite a piece of the glass tube and swallowed it.

When its mother went into the room she saw that the child's mouth was bleeding, and then discovered, to her horror, that the baby had swallowed a lot of glass.

This happened about half past 8 o'clock in the evening. I did not see the child until 10 o'clock ; it was sleeping quietly. Its lips and tongue had been but slightly cut. On inquiry, I learned that the child was "full of milk."

Now we come to the treatment. I imagine I hear a number of your readers say, "What did you do ?" I simply did nothing at all.

The next morning its bowels moved, and at noon again. In the second passage were seventy pieces of glass of different sizes ! Its bowels did not move again until the second day, when I ordered an injection of warm water and soap ; after the third injection the bowels moved, and we found four more pieces of glass—seventy-four pieces in all. Some of the pieces were as sharp as a needle. It is now one week since it swallowed the glass, and it has remained perfectly well the entire time.

My reason for not giving an emetic or a cathartic was that I did not think it advisable to irritate the alimentary tract and thus run the risk of having the bowels severely cut.

As I am a young member in the profession, I would like to know if such accidents are common—to swallow such an enormous amount of glass and create no disturbance at all ? Does not the swallowing of glass in any quantity generally prove fatal ?

I would like to hear from the older brethren on the accidental swallowing of foreign substances.

The pieces of glass varied from the size of a pin's head to three-eighths of an inch in length. Most of the pieces were long, slender and very pointed.

Dr. Formad says that no pathologist believes diptheria and croup to be identical diseases, but merely that the exudation in each is the same. While the exudation is similar in composition, there is this difference: the croupous membrane lays upon the mucous membrane, and the diptheric membrane extends down into it. The doctor makes the following common-place, but impressive, comparison: The croupous membrane is to the mucous membrane as a nail lying upon a table, while the diphtheritic membrane is to the mucous coat as a nail driven into the woodwork of a table. Croupous membrane can be readily separated from the mucous coat, while diphtheritic membrane is separated with difficulty, and leaves a bleeding surface.—*Medical World.*

**A New Preparation of Mercury.**—A new preparation, to which the name *hydrargyrum tannicum oxydulatum* has been given, is described by its sponsor, Dr. G. Lustgarter ("Wien. med. Wochensch," 1884, Nos. 11 to 14), as a dark, yellowish-green, odorless and tasteless powder, which does not change by keeping, and is easily assimilated when administered by the mouth. The dose is one-tenth of a gramme, and is given with milk-sugar in capsules, one-half to one hour after meals. The iodide of potassium, it is stated, cannot be safely used at the same time, on account of the danger of too large an amount of the iodide of mercury being formed. It is asserted of this preparation that it is not liable to cause stomatitis or salivation, nor to give rise to any trouble in the digestive tract. Its action in syphilis, in the few cases in which it was tried, is described as favorable.

**Iodide of Starch in Treatment of Intermittent Fever.**—Samuel R. Olliphant, M. D., New Orleans, writes: The first patient to whom I gave iodide of starch was a young man who had been under the treatment of several good physicians, and had taken, I suppose, nearly every known antiperiodic. But the chills would return every three weeks. I prescribed teaspoonful doses of the iodide three times a day. He passed over his time without any symptoms of a return, but subsequently had another chill and fever, and was again given the iodide. He was under my observation for several months after, and showed no disposition to relapse.—*Med. and Surg. Rep.*

Dr. R. H. Johnson (Medical Review) claims that tannin is a specific for carbuncles. The dry powder is sprinkled on the carbuncle; after a while it is washed off with Castile soap, and tannin reapplied. The application is not accompanied with pain, and the carbuncle assumes a healthy condition, and repair gradually takes place.—*Ibid.*

**Morphine Hypodermic Injections for Infantile Convulsions.**—Dr. C. C. P. Clark commends this treatment very highly, and reports cases to coroborate his views. Dr. Hughson, of Sumter, S. C., reports, in a later number of the same journal, a successful case similarly treated.—*Am. Jour. of Obstetrics.*

**The International Medical Congress of 1887.**—The General Committee for the organization of the next International Medical Congress, consisting of twenty-five members, held its first general meeting in Washington City, November 29, 1884. Fifteen members were present and answered to the roll-call. An executive committee of seven members was appointed, consisting of the president, general secretary, and treasurer, with Drs. S. C. Busey, of Washington, Christopher Johnson, of Baltimore, I. Minis Hays, of Philadelphia, and A. Jacobi, of New York. A finance committee of five members was directed to be appointed by the president, subject to the approval of the executive committee. A liberal basis of representation for the election of delegates to the Congress by the various medical society organizations in this country, was adopted, and the work of the Congress facilitated by the establishment of eighteen sections. The selection of officers of these several sections was not completed, but left in the hands of the executive committee. If the error of selecting these mainly from the few large cities of the Eastern and Middle States is not committed, we think the preliminary work of organization will have been placed on a good basis, with a fair prospect that the proposed Congress will receive the enthusiastic support of the profession of the whole country.—*Jour. Amer. Med. Association.*

Following closely upon the discovery that the hypodermic injection of theine—the active principle of tea—as a potent antidote in opium poisoning, comes the report that nitrite of amyl is still more efficient. A man who had taken two ounces of laudanum was resuitated after pulsation at the wrist had ceased, respiration fallen to six per minute, profound coma supervened, and the extremities became cold and cyanotic, by putting to his *nostrils* a napkin upon which thirty drops of the nitrite had been sprinkled. After two inhalations had been given he looked up and asked what was the matter.—*Medical World.*

The itching of urticaria is frequently relieved by a strong solution of bicarbonate of soda, a few drops of balsam copaiba on a lump of sugar, or in capsule, relieves sometimes. The bicarbonate of soda is mentioned as an almost certain remedy in poisoning by rhus toxicodendrum.—*National Druggist.*

**Electric Treatment of Perimetritis.**—Dr. Arostoli, of Paris, described his methods to the International Congress of Medical Sciences. He says that intra-uterine faradization, weak currents long continued, gives most excellent results in acute as well as chronic metritis and perimetritis.—*Le Progres Medical* (*Translation.*)

Dr. Carl Sieler, of Philadelphia, recommends tincture of benzoin in the treatment of frosted feet and chapped hands. It is painted over the parts. Oil may be applied to prevent the clothing from adhering.—*Louisville Med. News.*

